# Supplementary material for: Effect of whole-body vibration combined with exercise therapy on jump-landing stability after ACL reconstruction: A randomized controlled trial
Source: PLoS One. 2026 Feb 10;21(2):e0341553. doi: 10.1371/journal.pone.0341553 (PMC12890175; doi:10.1371/journal.pone.0341553)
Supplement: S1 Text — (ZIP) [file pone.0341553.s001.zip › Supporting Information/S1 Text.docx]

Introduction

Anterior cruciate ligament (ACL) rupture is one of the most common sports-related injuries. The incidence of ACL injury is estimated at approximately 70 per 100,000 individuals annually, with the cost of surgical reconstruction reaching up to $7.6 billion per year [1]. Moreover, individuals with ACL injury are three to five times more likely to develop knee osteoarthritis compared to healthy individuals [2].

The ACL is a dense band of connective tissue extending from the femur to the tibia, playing a critical role in stabilizing the knee joint against anterior tibial translation and rotational forces [3]. It contributes to both mechanical and functional stability of the joint. Functional stability of the lower limb depends on the complex interaction between afferent signals from mechanoreceptors in the skin, joints, ligaments, tendons, and muscles, and efferent signals from the central nervous system to alpha motor neurons, extrafusal muscle fibers, and muscle spindles. Consequently, ligament injury can impair proprioception, postural stability, strength, and neuromuscular control, all of which may compromise functional joint stability.

Therefore, ACL reconstruction aims to restore both mechanical and functional stability. However, evidence shows that even after surgery, full restoration of functional stability and return to pre-injury sports participation is not guaranteed. Approximately two-thirds of athletes do not return to their pre-injury level of sport one year after reconstruction, and only about 65% return to that level after an average of 3.5 years. Furthermore, only 36% continue participating in their primary sport seven years post-surgery. Among those who do return to sport, up to 29% may experience a re-injury of the same or contralateral ACL due to residual strength or performance deficits [4]. Additionally, studies have shown that even athletes who resume sports activities after ACL reconstruction often exhibit deficits in postural stability [5]. Poor postural stability has been identified as a key risk factor for ACL re-injury [6]. Hence, rehabilitation programs aiming to improve postural stability may help reduce the risk of re-injury in this population.

Various rehabilitation interventions—both open and closed kinetic chain exercises—have been proposed for individuals with ACL injury or reconstruction, with the goal of facilitating a safe return to sport. One such intervention is whole-body vibration (WBV), which is increasingly used in rehabilitation. WBV applies consistent vertical mechanical oscillations (30–50 Hz), stimulating various biological systems and potentially inducing physiological changes at multiple levels. These include activation of cutaneous receptors, muscle spindles, joint mechanoreceptors, and the vestibular system, as well as modulation of brain activity and neuroendocrine responses [7].

Evidence suggests that WBV can influence neuromuscular function and postural stability in individuals post-ACL reconstruction [1, 7–11]. Of five studies examining WBV effects on postural stability, only one reported no positive effects—likely due to the use of a single intervention session. This suggests that one session may be insufficient to induce meaningful neuromuscular adaptations. WBV appears to enhance the sensitivity and excitability of muscle spindles, which may reduce postural muscle response latency and lower the recruitment threshold of motor units [1]. However, in all previous studies, postural stability has been assessed using static measures, which evaluate a person’s ability to maintain their center of mass within the base of support.

While useful, these static tests may not sufficiently challenge the neuromuscular system to reveal functional deficits, especially in athletes. In contrast, dynamic postural stability—defined as the ability to maintain balance while transitioning from dynamic to static states—is more relevant for this population, as it better reflects the demands of sports participation. Tasks such as jump-landing may serve as more appropriate functional challenges for evaluating dynamic stability [6, 12, 13].

Static balance tests often fail to replicate the complexity of sport-specific or even daily functional tasks, and due to their simplicity, may not reveal underlying deficits. On the other hand, tasks like jump-landing more closely resemble sport-specific movements, during which most ACL injuries occur [12, 14]. In fact, over 70% of ACL injuries happen during non-contact activities such as landing, pivoting, or cutting [15]. Therefore, it has been suggested that adequate postural stability during high-risk movements like multidirectional jumping and cutting may be essential for a safe return to sport [3]. Some researchers argue that objective assessment of dynamic postural stability should be included in return-to-sport decision-making [6].

Two commonly used parameters for assessing dynamic postural stability are the Dynamic Postural Stability Index (DPSI) and Time to Stabilization (TTS). These parameters measure how well balance is restored following a dynamic-to-static transition and reflect functional neuromuscular control, particularly when derived from jump-landing tasks. Both DPSI and TTS have been shown to differentiate individuals with ACL injury or chronic ankle instability from healthy controls [12, 16–19]. Notably, TTS has been identified as a predictor of future ACL injury: each additional second in stabilization time during jump-landing may triple the risk of ACL rupture [20]. While TTS provides valuable information, DPSI has been shown to offer greater repeatability and accuracy [13]. Therefore, using both measures may provide a more comprehensive evaluation of dynamic postural stability.

Despite the growing use of WBV in sports rehabilitation and the recognized importance of dynamic postural stability during jump-landing, no study to date has examined the effect of WBV on these outcome measures. Thus, the central research question of this study is:
Can adding WBV to routine exercise improve dynamic postural stability during jump-landing more effectively than exercise alone in athletes post-ACL reconstruction?

Innovation

While existing studies suggest that WBV may enhance neuromuscular function and postural stability following ACL reconstruction, they have relied exclusively on static balance measures. These may not adequately challenge the neuromuscular system, particularly in athletes. In contrast, dynamic postural stability is more ecologically valid for sports settings, and jump-landing tasks more closely mimic the mechanisms of ACL injury. Given that most ACL injuries occur during high-speed, multi-directional movements, evaluating dynamic postural stability in this context is not only relevant but necessary. Despite this, no previous studies have explored the effectiveness of WBV on dynamic postural stability during jump-landing in athletes post-ACL reconstruction, marking a critical gap in the literature.

Practical Objectives

If WBV combined with exercise yields superior improvements in dynamic postural stability, it can be recommended as a cost-effective and functional rehabilitation approach in clinical settings. Conversely, if no significant difference is found between groups, it would suggest that routine exercise alone may suffice, potentially saving time and resources.

Study Design
Randomized, parallel-group clinical trial

Methods Summary

Inclusion Criteria:

The inclusion criteria were as follows: (1) participants aged 18 to 40 years with a history of ACL reconstruction for over six months; (2) the operated limb must be unilateral and dominant (used to kick a ball); (3) recreationally active (engaging in sports involving jumping like volleyball, basketball, soccer, or handball at least three times a week for more than 30 minutes each session)^30^; (4) returned to sports activities with medical clearance and completed rehabilitation; (5) no pain, inflammation, or limited range of motion in the knee; (6) no contraindications to WBV (e.g., pregnancy, acute thrombosis, severe cardiovascular problems, pacemaker, discopathy, spondylosis, severe diabetes, epilepsy, acute infection, severe migraine, tumor, and kidney stones) ^17^; and (7) no history of surgery or traumatic injuries to the contra-lateral limb.

The exclusion criteria included: (1) inability to perform the single-leg jump-landing task despite multiple practice attempts during the familiarization phase, indicated by consistent failure to maintain postural stability on the test limb and repeated demonstration of one or more of the following errors— involvement of the non-landing limb during takeoff or landing, additional hopping after landing, or excessive movement of the arms, trunk, or non-landing limb; (2) absence from two consecutive or three non-consecutive therapy sessions; and (3) unwillingness to continue participation.

Primary and Secondary Outcome Measures

*Pre- and post-intervention assessments:*

Laboratory Measures:

- Dynamic Postural Stability Index (DPSI) in anterior-posterior, medial-lateral, vertical, and composite directions during diagonal jump-landing
- Time to Stabilization (TTS) in anterior-posterior, medial-lateral, and composite directions during diagonal jump-landing

Clinical Measures:

- Limb Symmetry Index (LSI) in the 6-Meter Timed Hop (6MTH) Test
- Y-Balance Test

Intervention
Following baseline assessment, participants will be randomly assigned to one of two groups:

1. Control Group – Exercise only
2. Intervention Group – WBV + Exercise

Both groups will undergo a 12-session intervention over four weeks (3 sessions per week).

Sample Size
Based on the mediolateral TTS variable and data from Adelman et al. on the effects of WBV on dynamic stability in individuals with ankle instability, a sample size of 14 participants per group is estimated to detect pre-post differences [26].
