# Supplementary material for: Effect of whole-body vibration combined with exercise therapy on jump-landing stability after ACL reconstruction: A randomized controlled trial
Source: PLoS One. 2026 Feb 10;21(2):e0341553. doi: 10.1371/journal.pone.0341553 (PMC12890175; doi:10.1371/journal.pone.0341553)
Supplement: S1 Text — (ZIP) [file pone.0341553.s001.zip › Supporting Information/S2 Text.docx]

**مقدمه**

پارگی رباط صلیبی قدامی (ACL) یکی از شایع‌ترین آسیب‌های ورزشی است. میزان بروز آسیب ACL حدود ۷۰ مورد در هر ۱۰۰,۰۰۰ نفر در سال برآورد شده و هزینه بازسازی جراحی آن سالانه تا ۷.۶ میلیارد دلار می‌رسد [1]. علاوه بر این، احتمال ابتلا به استئوآرتریت زانو در افراد دچار آسیب ACL سه تا پنج برابر بیشتر از افراد سالم است [2].

رباط صلیبی قدامی نواری متراکم از بافت همبند است که از فمور تا تیبیا امتداد دارد و نقش حیاتی در پایداری زانو در برابر جابه‌جایی قدامی تیبیا و نیروهای چرخشی ایفا می‌کند [3]. این رباط هم در پایداری مکانیکی و هم در پایداری عملکردی مفصل نقش دارد. پایداری عملکردی اندام تحتانی وابسته به تعامل پیچیده بین سیگنال‌های آوران از گیرنده‌های پوستی، مفصلی، لیگامانی، تاندونی و عضلانی و سیگنال‌های وابران از سیستم عصبی مرکزی به نورون‌های حرکتی آلفا، فیبرهای عضلانی خارج‌دوکی و دوک‌های عضلانی است. در نتیجه، آسیب به لیگامان می‌تواند حس عمقی، پایداری پاسچرال، قدرت و کنترل عصبی‌عضلانی را مختل کند که همگی ممکن است پایداری عملکردی مفصل را به خطر اندازند.

بنابراین، هدف از بازسازی ACL، بازگرداندن هر دو جنبه مکانیکی و عملکردی پایداری است. با این حال، شواهد نشان می‌دهد که حتی پس از جراحی نیز بازگشت کامل عملکرد و حضور در سطح ورزشی پیش از آسیب تضمین‌شده نیست. تقریباً دو سوم ورزشکاران تا یک سال پس از بازسازی به سطح ورزشی پیشین خود بازنمی‌گردند و تنها حدود ۶۵٪ پس از میانگین ۳.۵ سال به آن سطح بازمی‌گردند. افزون بر این، فقط ۳۶٪ از ورزشکاران تا هفت سال پس از جراحی به فعالیت در رشته اصلی خود ادامه می‌دهند. در میان کسانی که بازگشت به ورزش دارند، تا ۲۹٪ ممکن است به‌دلیل باقی ماندن ضعف یا ناهنجاری عملکردی، دچار پارگی مجدد در همان یا پای مقابل شوند [4]. همچنین نشان داده شده است که حتی ورزشکارانی که پس از بازسازی ACL به فعالیت بازمی‌گردند، معمولاً دچار نقص در پایداری پاسچرال هستند [5]. پایداری ضعیف پاسچرال به‌عنوان یکی از عوامل خطر کلیدی برای آسیب مجدد ACL شناخته شده است [6]. بنابراین، برنامه‌های توان‌بخشی که با هدف بهبود پایداری پاسچرال طراحی می‌شوند، می‌توانند به کاهش خطر آسیب مجدد در این جمعیت کمک کنند.

روش‌های مختلف توان‌بخشی—شامل تمرین‌های زنجیره باز و بسته—برای افراد با آسیب یا بازسازی ACL پیشنهاد شده‌اند تا بازگشت ایمن به ورزش تسهیل شود. یکی از این روش‌ها **ویبره کل بدن** (WBV) است که کاربرد آن در توان‌بخشی به‌طور فزاینده‌ای در حال افزایش است. WBV با اعمال ارتعاشات مکانیکی عمودی پیوسته (۳۰ تا ۵۰ هرتز) باعث تحریک سیستم‌های زیستی مختلف و ایجاد تغییرات فیزیولوژیکی در سطوح گوناگون می‌شود. این تغییرات شامل فعال‌سازی گیرنده‌های پوستی، دوک‌های عضلانی، گیرنده‌های مکانیکی مفصل و سیستم دهلیزی، و نیز تعدیل فعالیت مغز و پاسخ‌های نورواندوکرین است [7].

شواهد نشان می‌دهد که WBV می‌تواند بر عملکرد عصبی‌عضلانی و پایداری پاسچرال در افراد پس از بازسازی ACL تأثیر بگذارد [1,7–11]. از میان پنج مطالعه بررسی‌کننده اثر WBV بر پایداری پاسچرال، تنها یکی نتایج مثبت گزارش نکرده که احتمالاً به‌دلیل استفاده از تنها یک جلسه مداخله بوده است. این امر نشان می‌دهد که یک جلسه ممکن است برای ایجاد سازگاری‌های عصبی‌عضلانی معنادار کافی نباشد. به نظر می‌رسد WBV حساسیت و تحریک‌پذیری دوک‌های عضلانی را افزایش داده، تأخیر پاسخ عضلات پاسچرال را کاهش داده و آستانه جذب واحدهای حرکتی را پایین می‌آورد [1]. با این حال، در تمام مطالعات پیشین، پایداری پاسچرال از طریق آزمون‌های **استاتیک** ارزیابی شده که توانایی فرد برای حفظ مرکز جرم در محدوده سطح اتکا را می‌سنجند.

اگرچه این آزمون‌ها مفید هستند، اما ممکن است سیستم عصبی‌عضلانی را به‌اندازه کافی به چالش نکشند تا نقص‌های عملکردی آشکار شوند، به‌ویژه در ورزشکاران. در مقابل، **پایداری پاسچرال دینامیک**—یعنی توانایی حفظ تعادل هنگام گذار از وضعیت دینامیک به استاتیک—برای این جمعیت مرتبط‌تر است، زیرا بازتاب بهتری از الزامات ورزشی دارد. وظایفی مانند **فرود از پرش**  (jump-landing) می‌توانند چالش‌های عملکردی مناسب‌تری برای ارزیابی پایداری دینامیک فراهم کنند [6,12,13[

آزمون‌های تعادل استاتیک اغلب نمی‌توانند پیچیدگی وظایف ورزشی یا حتی عملکردی روزمره را بازنمایی کنند و به‌دلیل سادگی ممکن است نقص‌های پنهان را آشکار نسازند. در مقابل، وظایفی مانند فرود از پرش بیشتر به حرکات ورزشی شباهت دارند—حرکاتی که بیشتر آسیب‌های ACL در آن‌ها رخ می‌دهد [12,14]. در واقع، بیش از ۷۰٪ از آسیب‌های ACL در فعالیت‌های بدون برخورد مانند فرود، چرخش یا تغییر جهت ناگهانی اتفاق می‌افتند [15]. بنابراین، پایداری پاسچرال کافی در حرکات پرخطر مانند پرش‌های چندجهته و تغییر جهت ناگهانی برای بازگشت ایمن به ورزش حیاتی است [3]. برخی پژوهشگران حتی پیشنهاد کرده‌اند که ارزیابی عینی پایداری دینامیک باید در تصمیم‌گیری برای بازگشت به ورزش گنجانده شود [6[.

دو شاخص متداول برای ارزیابی پایداری پاسچرال دینامیک عبارت‌اند از شاخص پایداری پاسچرال دینامیک (DPSI) و زمان تثبیت .(TTS) این شاخص‌ها توانایی بازگرداندن تعادل پس از یک انتقال دینامیک به استاتیک را اندازه‌گیری می‌کنند و کنترل عصبی‌عضلانی عملکردی را بازتاب می‌دهند—به‌ویژه زمانی که از وظایف فرود از پرش استخراج شوند. هر دو شاخص DPSI و TTS قادر به تمایز افراد با آسیب ACL یا ناپایداری مزمن مچ پا از افراد سالم هستند [12,16–19]. به‌طور خاص، نشان داده شده است که TTS می‌تواند پیش‌بینی‌کننده آسیب آینده ACL باشد: هر ثانیه تأخیر بیشتر در زمان تثبیت هنگام فرود از پرش ممکن است خطر پارگی ACL را تا سه برابر افزایش دهد [20]. با وجود ارزش اطلاعاتی TTS، شاخص DPSI قابلیت تکرارپذیری و دقت بالاتری دارد [13]. از این رو، استفاده هم‌زمان از هر دو شاخص می‌تواند ارزیابی جامع‌تری از پایداری دینامیک فراهم کند.

با وجود استفاده فزاینده از WBV در توان‌بخشی ورزشی و اهمیت شناخته‌شده پایداری پاسچرال دینامیک هنگام فرود از پرش، تاکنون هیچ مطالعه‌ای اثر WBV را بر این شاخص‌ها بررسی نکرده است. ازاین‌رو، پرسش اصلی پژوهش حاضر این است:
آیا افزودن WBV به تمرین‌های معمول می‌تواند پایداری پاسچرال دینامیک را هنگام فرود از پرش در ورزشکاران پس از بازسازی ACL به شکل مؤثرتری نسبت به تمرین صرف بهبود دهد؟

**نوآوری پژوهش**

اگرچه مطالعات موجود نشان داده‌اند که WBV ممکن است عملکرد عصبی‌عضلانی و پایداری پاسچرال را پس از بازسازی ACL بهبود بخشد، اما تمامی آن‌ها صرفاً از آزمون‌های تعادل استاتیک استفاده کرده‌اند که ممکن است سیستم عصبی‌عضلانی را به‌اندازه کافی به چالش نکشند—به‌ویژه در ورزشکاران. در مقابل، پایداری پاسچرال دینامیک از اعتبار بوم‌شناختی بالاتری برای محیط‌های ورزشی برخوردار است و وظایفی مانند فرود از پرش بیشتر با مکانیسم‌های واقعی آسیب ACL همخوانی دارند. با توجه به اینکه بیشتر آسیب‌های ACL در حرکات سریع و چندجهتی رخ می‌دهند، ارزیابی پایداری دینامیک در چنین زمینه‌ای نه‌تنها مرتبط بلکه ضروری است. با این حال، تاکنون هیچ پژوهشی اثر WBV را بر پایداری دینامیک هنگام فرود از پرش در ورزشکاران پس از بازسازی ACL بررسی نکرده است—که این موضوع شکاف مهمی در ادبیات علمی محسوب می‌شود.

**اهداف کاربردی**

اگر ترکیب WBV با تمرین منجر به بهبود بیشتر پایداری پاسچرال دینامیک شود، می‌توان آن را به‌عنوان روشی مؤثر، کم‌هزینه و کاربردی در توان‌بخشی معرفی کرد. در مقابل، اگر تفاوت معنی‌داری بین گروه‌ها مشاهده نشود، می‌توان نتیجه گرفت که تمرین به‌تنهایی کافی است و از صرف هزینه و زمان اضافی جلوگیری کرد.

**طرح مطالعه**

آزمایش بالینی تصادفی، دوسویه‌موازی

**خلاصه روش‌ها**

**معیارهای ورود:**

1. سن بین ۱۸ تا ۴۰ سال، با سابقه بازسازی ACL بیش از ۶ ماه؛
2. اندام جراحی‌شده باید یک‌طرفه و غالب (پایی که برای شوت‌زدن استفاده می‌شود) باشد؛

3. فعالیت ورزشی تفریحی شامل پرش (والیبال، بسکتبال، فوتبال یا هندبال) حداقل سه بار در هفته و بیش از ۳۰ دقیقه در هر جلسه؛

4. بازگشت به فعالیت ورزشی با تأیید پزشک و تکمیل برنامه توان‌بخشی؛

5. بدون درد، التهاب یا محدودیت حرکتی در زانو؛
6. بدون موارد منع مصرف WBV مانند بارداری، ترومبوز حاد، مشکلات شدید قلبی‌عروقی، ضربان‌ساز قلب، دیسکوپاتی، اسپوندیلوز، دیابت شدید، صرع، عفونت حاد، میگرن شدید، تومور و سنگ کلیه)؛

7. بدون سابقه جراحی یا آسیب تروماتیک در اندام مقابل.

**معیارهای خروج:**
1. ناتوانی در اجرای آزمون فرود تک‌پا با وجود تمرینات آشنایی، شامل عدم حفظ تعادل در اندام مورد آزمایش یا بروز خطاهایی مانند استفاده از پای مقابل، پرش اضافی پس از فرود یا حرکات زیاد دست‌ها و تنه؛
2. غیبت در دو جلسه متوالی یا سه جلسه غیرمتوالی؛
3. تمایل نداشتن به ادامه مشارکت.

**شاخص‌های پیامد اولیه و ثانویه:**
**ارزیابی‌های پیش و پس از مداخله:**

*آزمایشگاهی:*

- شاخص پایداری پاسچرال دینامیک (DPSI) در جهات قدامی–خلفی، داخلی–خارجی، عمودی و مرکب هنگام فرود مورب
- زمان تثبیت (TTS) در جهات مشابه هنگام فرود مورب

*بالینی:*

- شاخص تقارن اندام (LSI) در آزمون پرش ۶ متری زمان‌دار
- آزمون تعادل Y

**مداخله**

پس از ارزیابی اولیه، شرکت‌کنندگان به‌صورت تصادفی به یکی از دو گروه تخصیص می‌یابند:
۱. **گروه کنترل:** تمرین بدون WBV
۲. **گروه مداخله:** تمرین همراه با WBV

هر دو گروه در مجموع ۱۲ جلسه مداخله در طول چهار هفته (۳ جلسه در هفته) دریافت خواهند کرد.

**حجم نمونه**

بر اساس متغیر TTS **در جهت داخلی–خارجی** و با استناد به داده‌های مطالعه Adelman و همکاران درباره اثر WBV بر پایداری دینامیک در افراد دارای ناپایداری مچ پا، حجم نمونه‌ی لازم برای تشخیص تفاوت‌های پیش و پس از مداخله، ۱۴ شرکت‌کننده در هر گروه برآورد شد [26.[
