# Supplementary material for: Effect of whole-body vibration combined with exercise therapy on jump-landing stability after ACL reconstruction: A randomized controlled trial
Source: PLoS One. 2026 Feb 10;21(2):e0341553. doi: 10.1371/journal.pone.0341553 (PMC12890175; doi:10.1371/journal.pone.0341553)
Supplement: Attachment — Submitted filename: New Microsoft Word Document.pdf [file pone.0341553.s002.pdf]

Dear editors,

This randomized controlled trial (RCT) investigates a clinically relevant question using a task-specific outcome (jump-landing stability) in a post-ACL reconstruction population. The study is well-structured, registered, and assessor-blinded. However, critical methodological flaws, statistical inconsistencies, and overinterpretations compromise its validity and clinical interpretability. Below is a line-by-line, rigorous scientific critique with specific comments, contradictions, and mandatory corrections.

Title

Comment:

- The title is somewhat long and could be more concise. The phrase *"for improving jump-landing stability post-ACL reconstruction"* is grammatically correct but stylistically heavy.

Abstract

- The effect size (η^2) is not mentioned; this is essential in RCTs.
- The phrase *"clinical tests"* is vague; specify the tools (e.g., Y-Balance Test and Limb Symmetry Index).
- The final sentence *"suggesting enhanced neuromuscular control…"* overstates the findings; it should read: *"…suggesting potential enhancement in neuromuscular control…"*
- The phrase *"TTS significantly improved"* is misleading, as TTS is a time-based measure where lower values represent better performance. Therefore, the correct expression should be *"TTS significantly decreased."* Use this phrasing consistently throughout the manuscript to ensure conceptual accuracy.
- The text reports *"$P < 0.017$"* for YBT/LSI, implying a Bonferroni correction for three YBT directions. However, the Results section (Line 359) reports *"$P \leq 0.016$."* This inconsistency must be addressed by standardizing the threshold to $P < 0.016$ or by providing a clear justification for using 0.017.
- The statement *"routine exercise alone may be sufficient"* overgeneralizes the findings. Although both groups improved significantly ($P < 0.001$), this reflects descriptive equivalence rather than evidence of sufficiency. Rephrase the sentence as: *"No additional benefit was observed from WBV compared to routine exercise."*

Introduction

- The opening section (lines 58–70) is overly descriptive with many statistics that do not directly support the research gap. Consider condensing it.

- In line 93, *"An important methodological limitation"* has a capitalization error ("An" should start a new sentence or be connected grammatically).
- The research gap should be explicitly stated at the end, for example: *"However, the effect of WBV on dynamic postural stability during sport-specific tasks such as jump-landing remains unclear."*

- Reviews fatigue-induced changes in neuromuscular/biomechanical variables post-ACLR, showing similar responses to controls but persistent deficits—aligns with Lines 71–78 (re-injury risk) and 98–100 (dynamic tasks underestimate deficits). The authors may cite the following reference in lines 71–78 or 98-100: *Effect of Fatigue on Neuromuscular and Biomechanical Variables After Anterior Cruciate Ligament Reconstruction: A Systematic Review. For example,* Persistent neuromuscular deficits, exacerbated by fatigue [cite],
- "Effect of Fatigue on Neuromuscular and Biomechanical Variables After Anterior Cruciate Ligament Reconstruction: A Systematic Review"
- Discussion (Lines 374–559) : This reference supports the discussion on quadriceps inhibition and coordination (lines 388–396). Fatigue often mirrors patterns in healthy individuals but reveals ACL-specific vulnerabilities in landing kinetics. Suggested integration: *"Our TTS improvements may mitigate fatigue-related biomechanical alterations, as fatigue does not differentially impair ACLR patients vs. controls [cite], suggesting WBV enhances resilience under load."*
- Limitations (Lines 527–559): Since no fatigue protocol was tested in the current study, this reference can justify future research incorporating fatigue during jump-landing tasks. Suggested integration: *"Future work should incorporate fatigue protocols [cite] to assess WBV's durability in real-world athletic demands."*

The authors can used the following reference in different part of study; Psychosocial Interventions Seem to Reduce Kinesiophobia After Anterior Cruciate Ligament Reconstruction But Higher Level of Evidence Is Needed: A Systematic Review and Meta-Analysis

Introduction (Lines 58–132): Meta-analyses indicate that psychosocial interventions can reduce kinesiophobia, a major barrier to return-to-sport (Lines 66–71, 105–107). This aligns with the equivocal findings for WBV interventions (Lines 89–92). Kinesiophobia, as a key predictor of delayed or incomplete return-to-sport, may persist despite ACL reconstruction, highlighting the potential value of adjunctive therapies such as WBV for a more holistic recovery.

Discussion (Lines 374–559): Psychosocial factors influence neuromuscular control, which may explain why YBT and LSI improvements were similar across groups. While WBV effectively targets biomechanical stability, integrating psychosocial strategies could better address kinesiophobia, potentially enhancing return-to-sport rates.

Limitations (Lines 527–565): Evidence for long-term benefits of psychosocial interventions remains limited, and no long-term follow-up was conducted (Lines 538–549). Future research should combine WBV with psychological support in robust

longitudinal trials to evaluate the sustained impact on both neuromuscular performance and psychological readiness.

- Line 89–92: The manuscript states that da Costa et al. found "no significant effects," but this is misleading. That study examined the *acute*, single-session effects of WBV, whereas the present study investigates a *multi-session* intervention. The distinction between acute and long-term designs must be made explicit. Revise to: *"Unlike the acute, single-session WBV protocol used by da Costa et al., the current study implemented a multi-session intervention."*
- Line 97–98 :The phrase *"static tasks impose relatively low neuromuscular demands"* is inaccurate. Tasks performed on the Biodex platform at Levels 1–2 are inherently unstable and challenge balance substantially. This is a false generalization. Revise to: *"Static balance tasks on low Biodex stability levels may not fully replicate the multi-planar and high-velocity neuromuscular demands of sport."*
- Line 121–122: The text cites studies reporting reduced GRF [28] and shortened TTS [29] in healthy participants as if these findings apply to post-ACL populations. This is an overextension. These results highlight a research gap, not supporting evidence. Rephrase to: *"Preliminary evidence in healthy populations suggests that WBV may reduce GRF and TTS; however, its effects in post-ACL individuals remain unclear."*

Other studies that the authors could reference to strengthen the Introduction and Discussion include:

- Evaluation of Potential Factors Affecting Knee Function in Athletes After Anterior Cruciate Ligament Reconstruction
- Relationship Between Kinesiophobia and Vertical Ground Reaction Force in Anterior Cruciate Ligament Reconstructed and Deficient Patients During Landing Task
- Hip Abductor and Adductor Muscles Activity Patterns During Landing After Anterior Cruciate Ligament Injury

Methods

- Explain the allocation concealment and blinding procedures. Who administered WBV? Was the therapist blinded to group assignment?
- The reported *effect size = 0.066* is quite small; justification is needed (details from the pilot study should be included).
- It is unclear why SI is treated as the primary outcome when the main results focus on TTS. Clarify this inconsistency.

- Repeated-measures ANOVA is appropriate, but assumptions (sphericity, homogeneity) should be reported.
- Technical details are accurate but require methodological references (e.g., *Wikstrom et al., 2005*).
- Frequency (Hz), amplitude (mm), and duration per session must be explicitly stated for reproducibility.
- Control group equivalence: Both groups performed "routine exercises," but intensity and duration may differ; a comparative table should be added.

- Line 530–536 : A major methodological limitation is the absence of a WBV-only group. Without this comparison, it is impossible to determine whether the observed effects result from WBV itself or from its interaction with routine exercises. This design prevents answering a key question: *Is WBV alone sufficient to produce similar improvements?*
- Line 175–178 : Although initiating WBV six months post-operation is justified for safety reasons, this timing misses the critical neuromuscular recovery window typically observed between 3 and 6 months after ACL reconstruction. This delay may reduce the intervention's potential impact on neuromuscular adaptation and functional restoration.

Line 231–232: The use of a 3-second window for TTS analysis is questionable. Although references [20,31] are cited, ACL injuries typically occur within less than 50 milliseconds [50]. A 3-second post-landing analysis may not accurately reflect the critical phase of dynamic stabilization relevant to ACL injury mechanisms. The authors should justify the ecological validity of this time frame or consider restricting the analysis to the initial 0–1 second after landing.

Line 252 : The equation $RVTTS = \sqrt{(MLTTS^2 + APTTS^2)}$ assumes that both TTS components share the same units and scale, which may not be valid. TTS values should be normalized—such as by jump height, body weight, or another standard measure—before vector summation. Without normalization, this calculation is mathematically and conceptually invalid.

Line 208–210 : The exclusive use of a diagonal jump is justified with reference to Patterson [9]; however, this choice limits generalizability. Forward jump-landing tasks are more commonly associated with ACL injury mechanisms. The authors should explain why forward or vertical jumps were excluded or provide a rationale for choosing the diagonal direction as representative of sport-specific demands.

Line 311: The protocol applies WBV before the main exercise session, but no rationale is provided for this sequencing. Delivering WBV first may induce fatigue or neuromuscular pre-activation that could confound subsequent performance in strength and balance tasks. The authors should justify this order or discuss its potential impact on training outcomes.

Table 3:The Week 4 protocol (50 Hz with nine exercises) represents a relatively high cumulative WBV exposure. Such intensity may increase the risk of neuromuscular fatigue, overuse, or diminishing adaptive returns. The authors should provide justification for this progression,

supported by safety guidelines or prior studies demonstrating the tolerability of similar dosing parameters.

-

Results

- Some results are redundantly presented in both text and tables — shorten the text to avoid repetition.

- *p*-values should be reported consistently (e.g., use $p < 0.001$ instead of $p = 0.001$).
- Add 95% confidence intervals for between-group differences.
- Interpretation should acknowledge that significant effects were found only for AP and resultant directions in TTS, not all directions. This nuance should appear in the
-
- Line 365–367: The Limb Symmetry Index (LSI) formula appears reversed. The manuscript uses *(Healthy limb time / Injured limb time)* × *100*, which incorrectly yields values greater than 100% when the injured limb performs better—a conceptual error. The standard and widely accepted formula is: *LSI = (Injured limb performance / Healthy limb performance)* × *100.* This formulation ensures that values below 100% correctly indicate performance deficits in the injured limb. All LSI values must be recalculated.
- 351–352 : ML and V TTS data omitted | Only "no significant effects" → raw means ± SD must be in table. Transparency violation.
- 339–340 : Effect size underreported | AP TTS: $\eta^2 = 0.568$ (WBV group) vs. 0.150 (control) → large difference, but not in abstract. |

Discussion.

Line 387–388: The statement suggesting that the findings *"may reflect quadriceps inhibition or coordination deficits"* is speculative and unsupported by the data. Since no EMG, strength, or neural inhibition measures were collected, such interpretations exceed the study's evidence base. These claims should be softened or removed to avoid overinterpretation.

Line 396–403: The citation of *Maghbouli et al.* as supportive evidence is inappropriate, as that meta-analysis examined torque outcomes rather than TTS. The connection between muscle torque improvements and time-to-stabilization is indirect at best. Clarify this distinction and avoid implying direct comparability between unrelated outcome measures.

Line 492–498: The statement *"extended TTS may compromise injury prevention"* suggests a causal relationship not established in this study. TTS is a correlational performance indicator, not a validated causal predictor of re-injury risk. Rephrase to emphasize association rather than

causation, e.g., *"Prolonged TTS may be associated with impaired dynamic stability, which has been linked to higher injury risk in prior research."*

Line 560–565: The claim that *"WBV is more effective than exercise alone"* is overstated. The observed benefit was limited to TTS outcomes, with no significant differences in SI, YBT, or LSI measures. The conclusion should be revised to accurately reflect the selective nature of the effect, for example: *"WBV yielded additional improvements in TTS compared with exercise alone, but no differences were observed in SI, YBT, or LSI."*
