# Supplementary material for: Effect of whole-body vibration combined with exercise therapy on jump-landing stability after ACL reconstruction: A randomized controlled trial
Source: PLoS One. 2026 Feb 10;21(2):e0341553. doi: 10.1371/journal.pone.0341553 (PMC12890175; doi:10.1371/journal.pone.0341553)
Supplement: Attachment — Submitted filename: Overall Evaluation.pdf [file pone.0341553.s005.pdf]

Overall Evaluation

This revised manuscript (R1) reports an RCT examining the effects of whole-body vibration combined with routine exercise on postural stability, balance, and physical performance in athletes after ACL reconstruction. The study is relevant, uses objective biomechanical outcomes, and includes pilot-based power calculations. Revisions addressed prior reviewer comments (sample size, TTS reporting, introduction). Some methodological and reporting issues remain, such as incomplete blinding details, unstandardized training load, and limited statistical reporting, but overall, the manuscript is well-improved and requires only minor revision before being suitable for publication.

Major Comments:

1. **lines ~180–200:** The description of blinding remains insufficient. While the authors indicate a single-blind design (assessor-blinded), it is unclear whether participants or WBV therapists were blinded and how this was achieved (e.g., sham or placebo WBV). In physical intervention RCTs, participant blinding is challenging but essential to reduce performance bias, especially for subjective outcomes such as Y-Balance Test scores. Allocation concealment is not explicitly described—who generated the random sequence and how was it concealed from recruiters (e.g., sealed envelopes or third-party randomization)? Please provide the exact protocol and discuss the risk of bias using RoB 2 in the limitations. Without this, internal validity is compromised.
2. **lines ~450–500:** The age range (18–40) is justified for homogeneity, but the sample is mostly young (~25 years) elite athletes, limiting applicability to older ACLR patients or recreational athletes. Discuss how age-related neuromuscular declines (e.g., proprioception) might interact with WBV. Single-center design and lack of sex stratification (despite sex differences in ACL injury) are weaknesses—add in limitations and suggest multicenter studies.
3. **, lines ~400–450:** Statistically significant TTS reductions are reported, but clinical relevance without minimal detectable change (MDC) thresholds for ACLR patients may be overstated. MDC = 123.8 ms comes from healthy individuals—how is this applied here? Provide context: e.g., "TTS reduction of 128.99 ms exceeds MDC, indicating meaningful improvement, though confirmation in ACLR populations is needed." Avoid speculative language like "enhanced neuromuscular control" without direct measurement (e.g., EMG).

Minor Comments:

1. One of the most important methodological limitations is the absence of a control group receiving only WBV; without this group, it is impossible to determine whether the observed improvement in TTS is due to WBV itself or a synergistic effect of its combination with exercise.
2. Although this period is reasonable for safety, the 3- to 6-month post-surgery phase is recognized as the "golden window" for neuromuscular rehabilitation.
3. Starting WBV after 6 months may have missed the optimal opportunity to induce neuromuscular changes, and therefore the effects of WBV might appear less than potentially possible.
4. The lack of EMG use during landing, failure to measure muscle strength or muscular coordination, and the absence of an assessment of kinesiophobia or other psychological factors represent a significant gap in interpreting the underlying mechanisms.
5. An improvement in TTS might be due to a reduction in fear of movement or increased self-confidence, which WBV could indirectly influence, but this aspect was not investigated.
6. This may limit statistical power to detect smaller differences, especially in outcomes like SI, which did not show a significant change.
7. This study only examined short-term effects (immediately after the intervention); the lack of a 6- or 12-month follow-up to assess the sustainability of improvements, return-to-sport rate, or risk of re-injury is a major clinical weakness.
8. Does the improvement in TTS translate into a reduced risk of re-injury? Without follow-up, there is no answer to this question.
9. The landing task was performed under non-fatigued conditions, whereas many ACL injuries occur in fatigued states.
10. Assessing TTS under fatigued conditions could significantly increase the clinical value of the findings.
11. In the discussion section, there is a strong emphasis on the improvement in TTS, yet no improvement was observed in SI.
12. SI has higher reliability (ICC: 0.96) compared to TTS; the lack of improvement in SI despite improvement in TTS requires a more precise interpretation: does this TTS improvement truly indicate improved movement quality, or is it merely a temporal change without actual improvement in real control?

- Line 93: fix capitalization in “An important methodological limitation” → “an important methodological limitation” if sentence is continuous. Ensure all figures/tables (e.g., force plate setup) include scales and legends. Check and update references (e.g., Slobounov et al., 2006 for age effects), verify DOIs.
