# Supplementary material for: Effect of whole-body vibration combined with exercise therapy on jump-landing stability after ACL reconstruction: A randomized controlled trial
Source: PLoS One. 2026 Feb 10;21(2):e0341553. doi: 10.1371/journal.pone.0341553 (PMC12890175; doi:10.1371/journal.pone.0341553)
Supplement: Attachment — Submitted filename: PONE-D-25-57068.docx [file pone.0341553.s003.docx]

Dear Editor

Thank you for choosing me as a review (Manuscript Number PONE-D-25-20449). The following revise should be performed for this study.

Although the design of the article is good, there are important issues that are overlooked in the protocol that need to be clarified.

1- Provide a study timeline.
2- In the control group, how was the initial intensity of the exercises (10RM) measured? You got 10RM with a specific method.
3- What was the basis for increasing the intensity of the exercises in the intervention group? What was the basis for changing the frequency and duration of vibration administration?
4- What has been the effectiveness of your protocol in improving proprioception? Has proprioception been assessed at all? With what method and with what test?

Sincerely Yours
