# Supplementary material for: Effect of whole-body vibration combined with exercise therapy on jump-landing stability after ACL reconstruction: A randomized controlled trial
Source: PLoS One. 2026 Feb 10;21(2):e0341553. doi: 10.1371/journal.pone.0341553 (PMC12890175; doi:10.1371/journal.pone.0341553)
Supplement: Attachment — Submitted filename: Reviewer 3.docx [file pone.0341553.s004.docx]

**Dear Reviewer 3**

We sincerely appreciate your thorough review of the manuscript and the valuable comments and suggestions provided. We believe that the manuscript has significantly improved as a result of incorporating your feedback, which has been highlighted in green throughout the text.

**Question 1:** Provide a study timeline.

**Answer 1:** We would like to clarify that we were unsure of the specific format you expected for the study timeline. In general, the study included participant recruitment (April 3, 2022, to August 21, 2023), baseline assessments, a 4-week intervention period consisting of 12 supervised sessions (three sessions per week), and post-intervention assessments performed within 24–48 hours after the final session. These elements are described in detail throughout the Methods section, and we have now highlighted them in green in the manuscript for easier reference. A flow diagram based on the CONSORT statement (Figure 1) also illustrates participants’ progress from enrollment to analysis.

**Question 2:** In the control group, how was the initial intensity of the exercises (10RM) measured? You got 10RM with a specific method.

**Answer 2:** We added the following text for clarification: “The initial training intensity was determined using a standardized 10-repetition maximum (10RM) testing protocol [1]. At first, participants performed a standardized warm-up consisting of 5–10 repetitions at approximately 40–50% of their perceived maximal effort for the target muscle group, followed by a 1-minute rest period before the main 10RM testing trial. The resistance was then progressively increased in small increments of 5–10%. During each trial, participants performed up to 10 repetitions with correct technique. The load was adjusted in subsequent trials—if more than 10 repetitions were completed, the weight was increased, whereas if fewer than 10 repetitions were achieved, the weight was decreased after adequate rest—until the maximum load that could be lifted for exactly 10 repetitions with proper form and full range of motion was identified as the individual 10RM. Rest intervals of 2–3 minutes were provided between trials to minimize fatigue, and testing of different muscle groups was separated by sufficient recovery periods to prevent cumulative fatigue [1].”

**Question 3:** What was the basis for increasing the intensity of the exercises in the intervention group? What was the basis for changing the frequency and duration of vibration administration?

**Answer 3:** The progression of exercise intensity was based on established principles of progressive overload, task specificity, and neuromuscular adaptation. Exercise difficulty was gradually increased across sessions by advancing from bilateral to unilateral tasks, increasing resistance, reducing the base of support, and introducing controlled perturbations. This graded progression was designed to continuously challenge the sensorimotor system while maintaining participant safety. The perturbation-based elements of the protocol were inspired by Eitzen et al. (2010), while the strengthening component was adapted from Moezy et al. (2008). Both studies reported no significant adverse effects, supporting the safety of these approaches in post-ACL rehabilitation.

With respect to whole-body vibration (WBV), the vibration frequency, number of platform positions, and total exposure time were progressively increased across sessions based on the protocol described by Moezy et al. (2008). In that study, vibration frequency progressed from 30 Hz to 50 Hz, and during the final three sessions participants received a total of 16 minutes of vibration at 50 Hz across nine platform positions, with no reported adverse effects. We adopted the same gradual progression model to allow sufficient time for neuromuscular adaptation and to minimize the risk of excessive fatigue or overuse injury.

All training sessions in the present study were conducted under close clinical supervision, and participants were continuously monitored for pain, discomfort, or abnormal fatigue. No adverse events, neuromuscular fatigue, or overuse injuries were reported during the intervention period. Taken together, the progression of both exercise intensity and WBV parameters was grounded in prior published evidence and implemented with strict safety monitoring.

These descriptions have now been added to Sections 2.6.1 (Control Group) and 2.6.2 (Intervention Group), respectively.

**“**The progression of exercise intensity was guided by established principles of progressive overload, task specificity, and neuromuscular adaptation. This graded progression was designed to continuously challenge the sensorimotor system while ensuring participant safety, allowing sufficient time for physiological adaptation, and minimizing the risk of excessive fatigue or overuse injury. The progression strategy was grounded in prior published evidence, particularly the protocols described by Eitzen et al. (2010) and Moezy et al. (2008), and was implemented under strict safety monitoring [2, 3]. All training sessions were conducted under close clinical supervision, and participants were continuously monitored for pain, discomfort, or abnormal fatigue. No adverse events, neuromuscular fatigue, or overuse injuries were reported during the exercise.”

“The vibration frequency, number of platform positions, and total exposure time were progressively increased across sessions in accordance with the protocol described by Moezy et al. (2008)[3]. This stepwise progression was designed to promote gradual neuromuscular adaptation while minimizing the risk of excessive fatigue or overuse injury. All sessions were conducted under close clinical supervision, with continuous monitoring for pain, discomfort, or abnormal fatigue. No adverse events were reported throughout the intervention period.”

Moezy A, Olyaei G, Hadian M, Razi M, Faghihzadeh S. A comparative study of whole body vibration training and conventional training on knee proprioception and postural stability after anterior cruciate ligament reconstruction. British journal of sports medicine. 2008;42(5):373-8. Epub 2008/01/10. doi: 10.1136/bjsm.2007.038554. PubMed PMID: 18182623. ,

Eitzen I, Moksnes H, Snyder-Mackler L, Risberg MA. A progressive 5-week exercise therapy program leads to significant improvement in knee function early after anterior cruciate ligament injury. The Journal of orthopaedic and sports physical therapy. 2010;40(11):705-21. Epub 2010/08/17. doi: 10.2519/jospt.2010.3345. PubMed PMID: 20710097; PubMed Central PMCID: PMCPMC3158986.

**Question 4:** What has been the effectiveness of your protocol in improving proprioception? Has proprioception been assessed at all? With what method and with what test?

**Answer 4:** Proprioception was not directly assessed as an outcome in the present study; therefore, the effectiveness of our protocol on proprioceptive function cannot be determined directly from our data. However, previous research provides strong evidence that both whole-body vibration (WBV) and training interventions can enhance proprioceptive acuity.

In individuals following ACL reconstruction, Moezy et al. (2008) demonstrated that WBV significantly reduced absolute joint repositioning error at 30° and 60° of knee flexion, as assessed using a Biodex dynamometer, indicating improved joint position sense [3]. In addition, two recent systematic reviews (2023) in other musculoskeletal conditions, including low back pain and ankle instability, reported that WBV interventions are associated with significant improvements in joint proprioception [4, 5].

Moreover, several components of our training protocol—including strength exercises, and perturbation-based training—are known to stimulate muscle spindles, joint mechanoreceptors, and central sensorimotor pathways. Evidence from neuromuscular training studies shows that such exercises enhance proprioceptive feedback, inter-muscular coordination, and postural regulation through continuous sensorimotor challenge [6] [7].

Although proprioception was not measured directly in our study, the combination of WBV and routine exercises provides a strong mechanistic basis to hypothesize that neurosensory adaptations may have occurred. Accordingly, we propose that the observed improvements in jump biomechanics and dynamic postural control may be at least partially mediated by enhanced proprioceptive integration. This interpretation has now been explicitly clarified in the Discussion with appropriate supporting references.
