# Supplementary material for: Effect of whole-body vibration combined with exercise therapy on jump-landing stability after ACL reconstruction: A randomized controlled trial
Source: PLoS One. 2026 Feb 10;21(2):e0341553. doi: 10.1371/journal.pone.0341553 (PMC12890175; doi:10.1371/journal.pone.0341553)
Supplement: Attachment — Submitted filename: Reviewer 2.docx [file pone.0341553.s006.docx]

**Dear Reviewer 2**

We sincerely appreciate your thorough review of the manuscript and the valuable comments and suggestions provided. We believe that the manuscript has significantly improved as a result of incorporating your feedback, which has been highlighted in red throughout the text. In addition, some comments that addressed overlapping or closely related issues were integrated and addressed together to avoid redundancy and improve clarity.

**Question 1:** **lines ~180–200:** The description of blinding remains insufficient. While the authors indicate a single-blind design (assessor-blinded), it is unclear whether participants or WBV therapists were blinded and how this was achieved (e.g., sham or placebo WBV). In physical intervention RCTs, participant blinding is challenging but essential to reduce performance bias, especially for subjective outcomes such as Y-Balance Test scores. Allocation concealment is not explicitly described—who generated the random sequence and how was it concealed from recruiters (e.g., sealed envelopes or third-party randomization)? Please provide the exact protocol and discuss the risk of bias using RoB 2 in the limitations. Without this, internal validity is compromised.

**Answer 1:** We revised this section to: “Participants were randomly assigned to either the intervention group (WBV plus routine exercise) or the control group (routine exercise alone) with a 1:1 allocation ratio. Random sequence generation was performed by an independent researcher using a web-based randomization service ([www.randomization.com](http://www.randomization.com)). Group allocation was concealed using sequentially numbered, opaque, sealed envelopes prepared by a third party not involved in participant recruitment, intervention delivery, or outcome assessment.

The study employed a single-blind (assessor-blinded) design. Interventions were delivered by a trained physiotherapist who was not involved in outcome assessment. Outcome measurements were performed by an independent assessor who remained blinded to group allocation to minimize detection bias. Blinding of participants was not feasible due to the perceptible mechanical stimuli produced by WBV, which cannot be convincingly replicated without an active platform, and no validated sham WBV protocol was available. Treating physiotherapists were necessarily aware of group allocation because they administered the intervention.”

Additionally, the following was added to the Limitations section: “Additionally, blinding of participants was not feasible due to the perceptible nature of WBV, which may have introduced performance bias in effort-dependent outcomes such as the YBT. Treating physiotherapists were necessarily aware of group allocation because they administered the intervention, which may also contribute to performance bias. This limitation is acknowledged in the RoB 2 assessment.”

**Question 2:** **lines ~450–500:** The age range (18–40) is justified for homogeneity, but the sample is mostly young (~25 years) elite athletes, limiting applicability to older ACLR patients or recreational athletes. Discuss how age-related neuromuscular declines (e.g., proprioception) might interact with WBV. Single-center design and lack of sex stratification (despite sex differences in ACL injury) are weaknesses—add in limitations and suggest multicenter studies.

**Answer 2:** We added the following to the Limitation section: “Furthermore, although the age range was restricted to control for confounding effects of aging, the sample consisted predominantly of young adults (mean age ≈32 years) who were recreationally active. Consequently, the findings may not be generalizable to older individuals following ACLR, to less physically active populations, or to elite athletes with substantially higher training loads and neuromuscular demands. In addition, the single-center design may limit external validity. Finally, sex-specific analyses were not performed despite well-documented sex differences in ACL injury risk and neuromuscular control. Future studies should adopt multicenter designs with broader age ranges and include participants across different activity levels, with sex-stratified analyses, to better elucidate how age-, sex-, and activity-related factors influence the effects of WBV following ACL reconstruction.”

Additionally, we revised the Participant section to: “The sample was restricted to individuals aged 18–40 years to control for age as a key confounding factor associated with declines in balance, postural stability, and neuromuscular control. With advancing age, postural sway increases, sensory integration slows, and proprioceptive as well as reflexive balance control deteriorate [31]. Such age-related neuromuscular changes may alter responsiveness to WBV, potentially affecting the magnitude and nature of postural and functional adaptations in older compared with younger individuals. By limiting the age range, we reduced the likelihood that age-related deficits would obscure or modify the observed effects of WBV on postural control and jump biomechanics.”

**Question 3:** **lines ~400–450:** Statistically significant TTS reductions are reported, but clinical relevance without minimal detectable change (MDC) thresholds for ACLR patients may be overstated. MDC = 123.8 ms comes from healthy individuals—how is this applied here? Provide context: e.g., “TTS reduction of 128.99 ms exceeds MDC, indicating meaningful improvement, though confirmation in ACLR populations is needed.”

**Answer 3:** These paragraphs are intended to convey your comment: “Although the statistical analysis showed significant improvements in TTS, it is important to distinguish statistical significance from clinical relevance. Statistical significance indicates that an observed effect is unlikely to be due to chance, whereas clinical relevance relates to whether the magnitude of change is meaningful in a practical or therapeutic context. Because minimal clinically important difference (MCID) values have not yet been established for TTS, the minimal detectable change (MDC) was used as a reference to help determine whether the observed changes exceeded measurement error and normal biological variability. MDC represents the smallest change that can be interpreted as a true improvement beyond random variation [57].

In this study, the intervention group demonstrated reductions in TTS of 262.2 ms in the mediolateral direction and 128.99 ms overall, both exceeding the MDC value of 123.8 ms previously reported in healthy individuals [58]. Although caution is warranted when extrapolating MDC thresholds derived from healthy populations to individuals following ACLR, this comparison suggests that the observed improvements are likely to represent changes beyond random measurement error and normal biological variability. Nevertheless, the clinical meaningfulness of these improvements should be interpreted cautiously, and future studies are required to establish ACLR-specific MDC or MCID values to more definitively determine the clinical relevance of TTS improvements following WBV-enhanced rehabilitation.”

**Question 4:** One of the most important methodological limitations is the absence of a control group receiving only WBV; without this group, it is impossible to determine whether the observed improvement in TTS is due to WBV itself or a synergistic effect of its combination with exercise.

**Answer 4:** This limitation has been added to the Limitations section: “One of the most important methodological limitations of this study is the absence of a WBV-only control group. Without such a group, it is not possible to determine whether the observed improvements in TTS and other outcomes are attributable solely to WBV, to the exercise program, or to a synergistic effect of the two interventions. Including a WBV-only group would have allowed for a clearer isolation of the independent effects of WBV and a more precise evaluation of its contribution to dynamic stability and postural control.”

**Question 5:** Although this period is reasonable for safety, the 3- to 6-month post-surgery phase is recognized as the "golden window" for neuromuscular rehabilitation.

Starting WBV after 6 months may have missed the optimal opportunity to induce neuromuscular changes, and therefore the effects of WBV might appear less than potentially possible.

**Answer 5:** This limitation has been added to the Limitations section: “Moreover, WBV was initiated six months post-surgery. While this timing was reasonable for safety considerations, it falls just after the critical 3- to 6-month post-ACLR period, often regarded as the “golden window” for neuromuscular rehabilitation. Initiating WBV after this period may have missed the optimal opportunity to maximize neuromuscular adaptations, potentially attenuating the observed effects of the intervention on functional recovery.”

**Question 6:** The lack of EMG use during landing, failure to measure muscle strength or muscular coordination, and the absence of an assessment of kinesiophobia or other psychological factors represent a significant gap in interpreting the underlying mechanisms. An improvement in TTS might be due to a reduction in fear of movement or increased self-confidence, which WBV could indirectly influence, but this aspect was not investigated. This may limit statistical power to detect smaller differences, especially in outcomes like SI, which did not show a significant change.

**Answer 6:** This limitation has been added to the Limitations section: “A notable limitation of this study is the absence of assessments targeting the underlying mechanisms of the observed improvements. Specifically, EMG recordings during dynamic tasks such as jump-landing were not performed, and muscle strength or muscular coordination was not measured, limiting our understanding of the neuromuscular adaptations induced by WBV. Additionally, psychological factors such as kinesiophobia (fear of movement), which are well-established barriers to return-to-sport and full functional recovery after ACLR, were not evaluated. As such, improvements in outcomes like TTS may have been influenced by reductions in kinesiophobia or increases in self-confidence, which WBV could potentially modulate; however, these factors were not assessed, limiting interpretation of their contribution to the observed effects. Future studies should include EMG recordings, muscle strength and coordination assessments, and evaluations of psychological factors to better elucidate the mechanisms by which WBV—alone or combined with other interventions—enhances neuromuscular performance and supports functional recovery.”

**Question 7:** This study only examined short-term effects (immediately after the intervention); the lack of a 6- or 12-month follow-up to assess the sustainability of improvements, return-to-sport rate, or risk of re-injury is a major clinical weakness. Does the improvement in TTS translate into a reduced risk of re-injury? Without follow-up, there is no answer to this question.

**Answer 7:** This limitation has been added to the Limitations section: “Second, the absence of long-term follow-up assessments limits our understanding of the sustainability and clinical relevance of the observed treatment effects. This study evaluated outcomes only immediately post-intervention, leaving unanswered whether improvements in TTS and other functional measures persist over time, translate into successful return-to-sport, or reduce the risk of re-injury. Future studies should include 6–12 month follow-ups to assess re-injury rates, functional outcomes, and the durability of WBV effects, clarifying the long-term clinical impact of adding WBV to standard exercise protocols.”

**Question 8:** The landing task was performed under non-fatigued conditions, whereas many ACL injuries occur in fatigued states. Assessing TTS under fatigued conditions could significantly increase the clinical value of the findings.

**Answer 8:** This limitation has been added to the Limitations section: “In the present study, jump-landing tasks were performed under non-fatigued conditions, limiting the applicability of findings to real-world sport scenarios where many ACL injuries occur under fatigue. Future studies should assess TTS and related outcomes during fatigued states to better evaluate the practical and clinical relevance of WBV for injury prevention in sport-specific contexts.”

**Question 9:** In the discussion section, there is a strong emphasis on the improvement in TTS, yet no improvement was observed in SI. SI has higher reliability (ICC: 0.96) compared to TTS; the lack of improvement in SI despite improvement in TTS requires a more precise interpretation: does this TTS improvement truly indicate improved movement quality, or is it merely a temporal change without actual improvement in real control?

**Answer 9:** We appreciate the reviewer’s comment regarding the apparent discrepancy between TTS and SI outcomes. The intervention group demonstrated reductions in TTS exceeding the minimal detectable change (MDC) previously reported in healthy individuals, suggesting that these improvements likely reflect true enhancements in postural control rather than mere temporal fluctuations.

The lack of significant change in SI, however, can be explained by the fact that SI and TTS assess different aspects of dynamic stability. SI reflects inter-limb symmetry, whereas TTS captures the temporal component of achieving postural stabilization after a jump-landing task. Therefore, participants may achieve faster stabilization without substantial changes in limb symmetry, accounting for the unchanged SI values. We recognize that TTS and SI measure complementary, but not identical, dimensions of postural control, and both should be considered when interpreting the results.

We added this section to the Discussion: “Although SI has higher reliability than TTS, the reductions in TTS exceeding the MDC suggest that these improvements likely reflect genuine enhancements in postural control rather than mere temporal fluctuations, even in the absence of significant changes in SI. This discrepancy can be explained by the fact that SI and TTS assess complementary but distinct aspects of dynamic stability: SI reflects inter-limb symmetry, whereas TTS captures the temporal component of achieving postural stabilization after a jump-landing task. Therefore, participants may achieve faster stabilization without substantial changes in limb symmetry, accounting for the unchanged SI values [20, 32]. Together, these measures provide a more comprehensive understanding of postural control adaptations, and both should be considered when interpreting the results.”

**Question 10:** Line 93: fix capitalization in “An important methodological limitation” → “an important methodological limitation” if sentence is continuous. Ensure all figures/tables (e.g., force plate setup) include scales and legends. Check and update references (e.g., Slobounov et al., 2006 for age effects), verify DOIs.

**Answer 10:** Thank you for your comment. Upon checking, the manuscript already uses “…an important methodological limitation…” with “an” in lowercase, so no change is needed. All images include relevant scales and legends. We added DOI for Slobounov et al.
